# Extreme Heat and COVID-19 in New York City: An Evaluation of a Large Air Conditioner Distribution Program to Address Compounded Public Health Risks in Summer 2020

**DOI:** 10.1007/s11524-022-00704-9

**Published:** 2023-02-09

**Authors:** Kathryn Lane, Lauren Smalls-Mantey, Diana Hernández, Siobhan Watson, Sonal Jessel, Darby Jack, Leanne Spaulding, Carolyn Olson

**Affiliations:** 1 Bureau of Environmental Surveillance and Policy, New York City Department of Health and Mental Hygiene, New York, NY USA; 2 Sociomedical Sciences, Columbia University, Mailman School of Public Health, New York, NY USA; 3 New York City Housing Authority, New York, NY USA; 4 WE ACT for Environmental Justice, New York, NY USA; 5 Department of Environmental Health Sciences, Mailman School of Public Health, Columbia University, New York, NY USA; 6 Mayor’s Office of Climate and Environmental Justice, New York, NY USA

**Keywords:** Extreme heat, COVID-19, Climate change adaptation

## Abstract

In summer 2020, New York City (NYC) implemented a free air conditioner (AC) distribution program in response to the threats of extreme heat and COVID-19. The program distributed and installed ACs in the homes of nearly 73,000 older, low-income residents of public and private housing. To evaluate the program’s impact, survey data were collected from October 2020 to February 2021 via mail and online from 1447 program participants and 902 non-participating low-income NYC adults without AC as a comparison group. Data were examined by calculating frequencies, proportions, and logistic regression models. Participants were 3 times more likely to report staying home during hot weather in summer 2020 compared to non-participants (adjusted odds ratio [AOR] = 3.0, 95% confidence interval [CI] = 2.2, 4.1), with no difference between groups in summer 2019 (AOR = 1.0, CI = 0.8, 1.3). Participants were less likely to report that 2020 hot weather made them feel sick in their homes compared to non-participants (AOR = 0.2, CI = 0.2, 0.3). The program helped participants—low-income residents and primarily people of color—stay home safely during hot weather. These results are relevant for climate change health-adaptation efforts and heat-health interventions.

**Supplementary Information:**

The online version contains supplementary material available at 10.1007/s11524-022-00704-9.

## Introduction

In spring 2020, New York City was the US epicenter of the SARS-CoV-2 (COVID-19) pandemic that had taken the lives of nearly 20,000 residents and resulted in the hospital admission of more than 70,000 people by April 15, 2020 [1]. At the time, no effective treatments were known or vaccines available. New Yorkers were advised to stay home as much as possible as part of social distancing guidelines to prevent viral transmission. Schools, many workplaces, and public spaces were closed, including those that would usually serve as cooling centers during extreme heat events.

In a typical summer, the City advises those at risk of heat-health problems to use their air conditioning (AC) if they have one, setting it to low-cool or 78 °F to save money and protect the electric grid. Those without AC are advised to seek out a cool place, such as a City-run cooling center, an air-conditioned public place, or the home of family or friends with AC. In spring and summer 2020, however, restrictions and closures of non-essential businesses due to the COVID-19 pandemic significantly limited these options for New Yorkers without AC.

In an ordinary year, heat exposure takes a tremendous toll on the health of New Yorkers, killing on average 350 residents a year from 2010 to 2018 by worsening chronic health conditions [2]. These deaths inequitably affect non-Latinx Black New Yorkers [3]. These inequities are rooted in systemic racism that limits economic opportunities, access to health care, and leads to neighborhood and housing disinvestment [4, 5].

AC is the best protection against the adverse health effects of extreme heat. Cities with a higher AC prevalence experience fewer heat-related deaths [6–8]. Among New Yorkers who died of heat stress (i.e. the death was recognized and coded as caused by heat) from 2010 to 2019, 71% died in un-air-conditioned homes [2]. Non-Latinx Black and low-income NYC residents, however, are much less likely to have AC [9]. In spring 2020, Black and Latinx New Yorkers also were more likely to hold jobs that did not allow remote work [10], resulting in higher levels of SARS-CoV-2 exposure, and extremely high COVID-19 mortality rates [11]. Older age and having chronic underlying conditions, such as kidney conditions, diabetes, and respiratory or heart disease, also increased risk of severe outcomes from both COVID-19 and heat-related illness [12–15].

In response to the combined threats of COVID-19 and extreme heat, on May 15, 2020, NYC announced the creation of the Get Cool NYC emergency program, managed by NYC Emergency Management (NYCEM), which distributed and installed nearly 73,000 free home air conditioners to low-income adults aged 60 and older who did not have a working home AC in both New York City Housing Authority (NYCHA) public housing and private housing. In June, the first month of operation, enrollment was limited to clients of means-tested benefit or City programs, such as senior service programs, because they were already age and income verified.

Beginning in late June, supportive housing partners working with the Department of Health and Mental Hygiene (DOHMH) and property managers of Section 8 voucher buildings working with the Department of Housing Preservation and Development Authority (HPD) began enrolling eligible residents in their programs. In addition, the Health Department invited several hundred community- and faith-based organizations to enroll clients through an agreement with the Department allowing the organizations to verify client eligibility. More than 100 organizations, including the three organizations working with the existing Be a Buddy climate resilience program, enrolled approximately 5000 people. New Yorkers could also call 311, the City government’s information and help service, to inquire about eligibility.

NYCHA is home to more than 350,000 New Yorkers in about 161,000 households in public housing, of which 41% are headed by an adult aged 62 or older and 91% are headed by someone who is Latinx or Black [16]. The average family size is 2.2 people and the average family income is $24,503 [17]. For the Get Cool program, NYCHA aimed to make AC installation available for all households that did not have a working AC with a resident aged 60 and older or with a resident of any age with a registered mobility impairment or dependence on lifesaving medical equipment in NYCHA records. Installed AC units belonged to NYCHA to facilitate ongoing maintenance; this also allowed the agency to waive the monthly AC surcharge it typically charges for resident-owned AC installation and use during the pandemic. NYCHA coordinated closely with the larger Get Cool program, but its AC installation and data management efforts were managed in-house.

Between June and September 2020, Get Cool led to the installation of more than 16,000 AC units in NYCHA homes and more than 56,000 in non-NYCHA homes. We performed an equity assessment of installations to better understand the neighborhood reach of the program. To evaluate the impact of receiving an AC, we surveyed Get Cool participants and a comparison group of non-participants about their experiences with hot weather during summer 2019 and 2020. The survey also asked about barriers to AC acquisition and use. An understanding of outcomes from this emergency program can inform future interventions to address increasing and intensifying heat due to climate change, particularly among people who are at increased heat-health risk.

## Methods

The study team included members from DOHMH, Columbia University’s Mailman School of Public Health, WE ACT for Environmental Justice (WE ACT), and NYCHA, with support from HPD and the New York City Department for the Aging (DFTA). AC installation rates were mapped to understand the spatial distribution of the program using Get Cool program AC installation data from NYCEM. Denominators were based on the number of adults 60 and older living below the federal poverty line from 2015 to 2019 American Community Survey population estimates. We also calculated AC installation rates by DOHMH heat vulnerability index (HVI) level and NYC Task Force for Racial Inclusion and Equity (TRIE) neighborhoods. The HVI estimates heat-health risk across NYC neighborhoods based on a statistical model that uses social and environmental factors, with levels 1 to 5 for lowest to highest risk [3, 18]. TRIE neighborhoods were designated in 2020 as those most impacted by COVID-19 and a high percentage of other health and socioeconomic inequities [19]. Rate denominators for HVI and TRIE areas were based on DOHMH population estimates for all adults aged 60 years and older in 2019, modified from US Census Bureau intercensal population estimates for modified zip code tabulation areas.

The survey included questions about household AC, where and how people kept cool during very hot weather in summer 2019 and 2020, feeling sick from heat while home, how the household was affected by COVID-19, barriers to using or purchasing AC in the past, energy insecurity, self-reported health status, and basic demographics. Participant and non-participant surveys were identical, except the participant survey included a question about Get Cool program satisfaction and references to receiving an AC. On both surveys, survey respondents had the option of writing brief free text responses to some questions.

During survey development, DOHMH convened a virtual meeting of about 80 partners from community-based organizations participating in the Get Cool program to describe evaluation plans and gather feedback on the draft survey. We tailored the survey for public housing residents, removing questions about energy insecurity, appropriate only for respondents paying utility bills directly, and household income. All survey respondents were offered a $10 gift card as an incentive.

### Survey Among Public Housing Residents

NYCHA sent the survey to 5300 heads of households who received ACs. Recipients were randomly sampled from the 8055 Get Cool records entered in the NYCHA database as of October 2020. Because NYCHA aimed to provide all its residents aged 60 and older with ACs, the comparison survey was sent to 5300 heads of household aged 53–59 (younger than eligibility age) who were not otherwise eligible for Get Cool and had no NYCHA record of an AC. All 5028 heads of households aged 54–59 without a registered AC were included in the sample and the remaining 272 people were randomly selected from 802 heads of household aged 53. Some de-identified administrative data, such as age, race/ethnicity, and whether electricity was paid directly, were included in the NYCHA dataset from which the Get Cool participant and comparison records were randomly sampled. These elements were linked with survey responses based on survey ID numbers.

The survey was mailed in English and Spanish with a pre-paid return envelope. The cover letter included directions for how to take the survey online in English, Spanish, Russian, Traditional Chinese, and Simplified Chinese. Respondents could take the survey from November 13, 2020, to February 21, 2021. Because comparison group letters were mailed 2 weeks late, a reminder letter was sent to the comparison group in December 2020, making it clear that they had more time to take the survey. The participant version of the survey included 24 questions and the comparison included 23 questions. De-identified paper survey responses were data entered by trained DOHMH staff.

### Survey Among Residents of Private Housing

The private housing survey was mailed to 3800 people who received Get Cool ACs, who were randomly sampled from 23,195 participants who agreed to follow-up contact at the time of AC installation. Surveys were distributed to a comparison group of 8329 non-eligible New Yorkers comprising the following: (1) HPD-managed Sect. 8 tenants aged 55–59 years (*n* = 5300); (2) DFTA clients not eligible for the program (*n* = 21); and (3) New Yorkers who called 311 to inquire about the program but were not eligible (*n* = 3008).

DOHMH contracted with ICF, an independent survey research firm, to mail the survey, as well as two reminder letters and two reminder emails (for those with an email address). Respondents could complete the survey in English or Spanish on paper with a pre-paid return envelope or in any of 7 languages online (Haitian-Creole and French, in addition to the languages available to NYCHA residents). Respondents could take the survey from October 9, 2020, to December 9, 2020. The participant version of the survey included 30 questions and the comparison included 29 questions. Trained ICF staff completed data entry.

### Statistical Methods

Data were processed and analyzed in SAS (SAS Institute, Cary, NC). Data from the public housing and private housing surveys were examined separately and then pooled together. The comparison group was restricted to respondents who reported not having a home AC in 2019. Data were examined by calculating frequencies, proportions, and logistic regression models. Missing data were excluded from analyses. Significance was assessed by examining magnitude of estimates and confidence limits.

This research was reviewed by the NYC DOHMH and Columbia University Institutional Review Boards and was determined to be exempt.

## Results

**Fig. 1:**
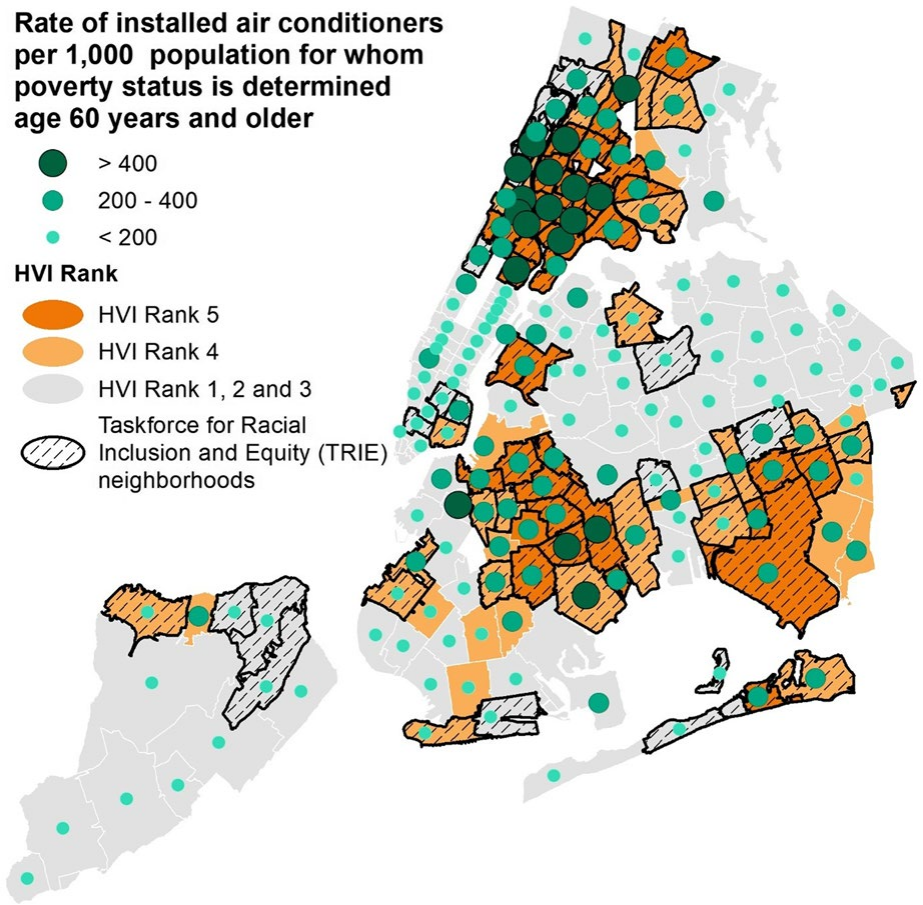
Rate of Get Cool AC installations by modified zip code tabulation area, heat vulnerability index (HVI) level, and NYC Task Force for Racial Inclusion and Equity (TRIE) neighborhoods designated as most impacted by COVID-19 and other health and socioeconomic disparities. Rate denominators are the number of adults 60 and older living below the federal poverty level. Rate numerators based on NYC Emergency Management Get Cool program AC installation data. Map created by Erika Poulsen, NYC Department of Health and Mental Hygiene

The Get Cool program reached every neighborhood of the city [20]. AC installation rates were higher in areas with more older adults living below the federal poverty level (see Fig. 1). More ACs were installed in neighborhoods ranking high on the DOHMH Heat Vulnerability Index [3] and with the greatest morbidity and mortality from COVID-19. Installation rates were more than twice as high in HVI 4 areas (47.7 per 1000 older adults) and nearly five times higher in HVI 5 areas (94.1 per 1000) compared to HVI levels 1–3 areas (20.4 per 1000, see Supplemental Table 1). Installations in TRIE areas were about four times higher compared to non-TRIE areas (68.1 installations vs. 18.1 per 1000).

Together, the surveys had a response rate of 15.9% (1447 of 9100 mailed) for participants and 13.8% (1885 of 13,629 mailed) for the comparison group using the American Association of Public Opinion Research response rate 2 definition, i.e., the number of returned complete and partial surveys divided by the total number people invited to take the survey. In the comparison group, 902 (48%) respondents reported not having AC in 2019 (*n* = 507 for the public housing survey, and *n* = 395 for the private housing survey) and were included in this analysis. Among the 2349 responses analyzed, 88% (*n* = 2056) of responses were in English, 11% (*n* = 266) in Spanish, 1% (*n* = 13) in Chinese-Traditional, and the remaining 1% were in Chinese-Simplified (*n* = 6), Russian (*n* = 7), and Creole (*n* = 1). Mailed surveys accounted for 74% (*n* = 1732) of responses.

Respondents were primarily people of color, with the participant group including more Latinx (39% versus 32%) and fewer non-Latinx Black residents (39% versus 54%) than the comparison (Table 1). About 60% reported not having an AC in 2019. One quarter of the comparison group was aged 60 or older. Seventy percent (95% confidence interval [CI]: 65, 75) of the private housing comparison and 67% (CI: 63, 70) of the private housing participant group reported a household income of less than $20,000 (data not shown). Chronic health conditions were prevalent in both groups, including hypertension, diabetes, and asthma or chronic obstructive pulmonary disease (COPD). In addition, 24% (CI: 22, 26) of participants and 29% (CI: 26, 32) of non-participants reported that they or someone in their household had anxiety, depression, or other mental health conditions.

**Table 1:** Demographic characteristics, health status, and COVID-19 effects on household for Get Cool participant and comparison group survey respondents in New York City, 2020

	Program participants		Comparison group	
Respondent characteristics	*n* = 1447	% (95% CI^d^)	*n* = 902	% (95% CI)
Race and ethnicity^a^				
Non-Hispanic Black	549	39 (36, 41)	480	54 (50, 57)
Non-Hispanic White	119	8 (7, 10)	46	5 (4, 7)
Hispanic, Latino, or Spanish	551	39 (36, 41)	283	32 (29, 35)
Non-Hispanic Asian or Pacific Islander	117	8 (7, 10)	47	5 (4, 7)
Other	28	2 (1, 3)	19	2 (1, 3)
Prefer not to say	57	4 (3, 5)	21	2 (1, 3)
*Missing*	*26*		*6*	
Home AC^b^				
No AC in summer 2019	819	57 (54, 59)	902	100
Age group^a^				
Under 60	109	8 (6, 9)	671	75 (72, 78)
60 plus	1304	92 (91, 94)	221	25 (22, 28)
*Missing*	*34*		*10*	
Household health conditions^c^				
Diabetes	595	41 (39, 44)	276	31 (28, 34)
Hypertension	915	63 (61, 66)	449	50 (47, 53)
Cardiovascular conditions (CVD)	324	22 (20, 25)	131	15 (12, 17)
Overweight or obese	302	21 (19, 23)	169	19 (16, 21)
Asthma or chronic obstructive pulmonary disease (COPD)	364	25 (23, 27)	244	27 (24, 30)
Physical mobility hardships	386	27 (24, 29)	156	17 (15, 20)
Chronic kidney disease	70	5 (4, 6)	31	3 (2, 5)
Electric medical equipment	113	8 (6, 9)	55	6 (5, 8)
Cognitive impairments	39	3 (2, 4)	19	2 (1, 3)
Anxiety, depression or other mental health conditions	350	24 (22, 26)	263	29 (26, 32)
None	145	10 (8, 12)	170	19 (16, 21)
Prefer not to say	75	5 (4, 6)	61	7 (5, 8)
*Missing*	*2*		*2*	
Household size (number of people)				
1	709	55 (52, 57)	466	54 (51, 58)
2	328	25 (23, 28)	231	27 (24, 30)
3	126	10 (8, 11)	83	10 (8, 12)
≥ 4	137	11 (9, 12)	78	9 (7, 11)
*Missing*	*147*		*44*	
General health status				
Excellent, Good, Very Good	598	42 (40, 45)	418	48 (45, 51)
Poor/Fair	815	58 (55, 60)	452	52 (49, 55)
*Missing*	*34*		*32*	
COVID-19 effect on household^c^				
Household member lost their job	279	19 (17, 21)	183	20 (18, 23)
Household member diagnosed with COVID-19	163	11 (10, 13)	68	8 (6, 9)
Household member hospitalized due to COVID-19	64	4 (3, 5)	21	2 (1, 3)
Household member passed away from COVID-19	67	5 (4, 6)	24	3 (2, 4)
Trouble providing food for family	241	17 (15, 19)	176	20 (17, 22)
Trouble paying rent or mortgage	254	18 (16, 20)	226	25 (22, 28)
Trouble providing/affording childcare	17	1 (1, 2)	19	2 (1, 3)
Trouble helping children with their schooling	44	3 (2, 4)	52	6 (4, 7)
Did not affect household	653	45 (43, 48)	374	41 (38, 45)
Other	194	13 (12, 15)	138	15 (13,18)

^a^Public housing respondent data uses head of household race/ethnicity and age from administrative data, while private housing race/ethnicity and age questions are self-reported

^b^Respondent reported not having a home AC in 2019

^c^Respondent could choose more than one option

^d^Confidence interval

Both groups were affected by COVID-19, with 11% (CI: 10, 13) of participants and 8% (CI: 6, 9) of non-participants reporting a household member diagnosed with COVID-19. Five percent (CI: 4, 6) of participants and 3% (CI: 2, 4) of the comparison group reported that a household member died. Some respondents wrote that even though they did not lose a household member or experience illness in the household, they were still deeply affected by the pandemic. “From all my childhood friends, I am the only one left with a living parent (who did not die of Covid). I am their relief, but it is trying for me,” wrote a respondent. About one-fifth of each group reported having a household member lose a job. “My job ended on March 15,” wrote one respondent. “I lost a substantial amount of my income.” Some respondents described experiencing fear and isolation because of COVID-19: “It took a toll on my mental state of mind. I was feeling depressed,” said a respondent. “It is a very scary time, and I am worried for myself and my relatives,” wrote another. “I can’t go to adult day care, can’t communicate with my friends, can’t get enough food on time,” wrote another respondent.

In summer 2020, more participants reported staying home during very hot weather than non-participants (Table 2: 89%, CI: 88, 91 versus 73%, CI: 70, 76 respectively). Among participants, 87% (CI: 85, 89) used AC to cool their homes during very hot weather in 2020 always, most of the time, or half the time (data not shown). Participants were also more likely to report staying home because they were comfortable there. In summer 2019, there was no difference in staying home between the groups (Table 2: 71%, CI: 69, 74 versus 69%, CI: 66, 72 respectively). Non-participants were more likely to go to a work location to cool off in 2020 (12% [CI: 10, 14] for the comparison group and 5% [CI :4, 6] for participant), possibly because the comparison group skewed younger, although the majority of both groups did not go to work to cool off. More than one-quarter of non-participants (27%, CI: 24, 30) reported that hot weather made them or someone in their household feel sick at home in summer 2020, compared to only 10% (CI: 9, 12) of participants. Only 7% (CI: 6, 9) of participants and 9% (CI: 7, 10) of non-participants reported going to a City-designated cooling center in 2020, with slightly higher rates of attendance in 2019 (17% of participants and 11% of non-participants).

**Table 2:** Experiences in summer 2020 and 2019 by Get Cool participant and comparison groups, New York City

	Program participants		Comparison group	
	*n*	% (95% CI^b^)	*n*	% (95% CI)
Location(s) to cool off in very hot weather (summer 2020)^a^				
Stayed home	1,293	89 (88, 91)	656	73 (70, 76)
Visited a neighbor/friend/family member’s house	130	9 (8, 10)	176	20 (17, 22)
Went to a work location	74	5 (4, 6)	106	12 (10, 14)
Went to a business (pharmacy, grocery store, barber shop, etc.)	135	9 (8, 11)	96	11 (9, 13)
NYC Cool Streets program (street closed to cars)	40	3 (2, 4)	22	2 (1, 3)
Went somewhere else outside (beach, park, stoop)	241	17 (15, 19)	231	26 (23, 28)
City-designated cooling center (public library, senior or community center)	105	7 (6, 9)	77	9 (7, 10)
Church, temple, mosque or other place of worship	66	5 (3, 6)	73	8 (6, 10)
Other	63	4 (3, 5)	72	8 (6, 10)
Main reasons for staying home (summer 2020)^a^				
Home was comfortable	1,026	79 (77, 82)	400	61 (57, 65)
Concerned about COVID/social distancing	973	75 (73, 78)	488	74 (71, 78)
Concerned about safety in the community	290	22 (20, 25)	172	26 (23, 30)
Childcare responsibilities	17	1 (1, 2)	12	2 (1, 3)
Working from home	37	3 (2, 4)	40	6 (4, 8)
Nowhere else to go	238	18 (16, 21)	148	23 (19, 26)
Limited transportation	68	5 (4, 6)	53	8 (6, 10)
Difficulty moving or walking	242	19 (17, 21)	95	14 (12, 17)
Not applicable/I did not stay home	18	1 (1, 2)	21	3 (2, 5)
Other	35	3 (2, 4)	46	7 (5, 9)
Location(s) to cool off in very hot weather (summer 2019)^a^				
Stayed home	1,032	71 (69, 74)	622	69 (66, 72)
Visited a neighbor/friend/family member's house	216	15 (13, 17)	198	22 (19, 25)
Went to a work location	89	6 (5, 7)	140	16 (13, 18)
Went to a business (pharmacy, grocery store, barber shop, etc.)	172	12 (10, 14)	120	13 (11, 16)
Went somewhere else outside (beach, park, stoop)	373	26 (24, 28)	255	28 (25, 31)
City-designated cooling center (public library, senior or community center)	243	17 (15, 19)	97	11 (9, 13)
Church, temple, mosque or other place of worship	125	9 (7, 10)	89	10 (8, 12)
Other	71	5 (4, 6)	64	7 (5, 9)
Main reasons for staying home (summer 2019)^a^				
Home was comfortable	792	77 (74, 79)	439	71 (67, 74)
Concerned about safety in the community	280	27 (24, 30)	192	31 (27, 35)
Childcare responsibilities	31	3 (2, 4)	17	3 (1, 4)
Working from home	34	3 (2, 4)	15	2 (1, 4)
Nowhere else to go	310	30 (27, 33)	200	32 (28, 36)
Limited transportation	116	11 (9, 13)	74	12 (9, 14)
Difficulty moving or walking	289	28 (25, 31)	111	18 (15, 21)
Not applicable/I did not stay home	40	4 (3, 5)	39	6 (4, 8)
Other	59	6 (4, 7)	55	9 (7, 11)
Household member feeling sick at home due to hot weather (2020)				
Yes	150	10 (9, 12)	241	27 (24, 30)
No	1,127	78 (76, 80)	521	58 (55, 61)
Not sure	170	12 (10, 13)	140	16 (13, 18)

^a^Respondents could pick more than one option

^b^Confidence interval

In logistic regression models including race/ethnicity and age, participants were three times more likely to stay home in 2020 during hot weather compared to non-participants (Table 3, adjusted odds ratio [AOR] = 3.0, CI: 2.2, 4.1), but there was no difference between groups in summer 2019 (AOR = 1.0, CI: 0.8, 1.3). Also adjusting for race/ethnicity and age, participants were less likely to report that hot weather made them feel sick in their homes (AOR = 0.2, CI = 0.2, 0.3). Overall, 91% of participants reported satisfaction with the Get Cool program.

**Table 3:** Univariate and multivariate predictors of staying home during summer 2020 and 2019 and feeling sick inside at home because of heat in 2020, New York City

		Private housing		Public housing		Pooled	
Outcome	Predictor	Univariate OR (95% CI)	Multivariate^a^ OR (95% CI)	Univariate OR (95% CI)	Multivariate^b^ OR (95% CI)	Univariate OR (95% CI)	Multivariate^a^ OR (95% CI)
Stay home 2020	Program participant vs non-participant	**4.2 (3.1, 5.8)**	**3.5 (2.5, 5.0)**	**2.4 (1.7, 3.3)**	**2.4 (1.7, 3.4)**	**3.1 (2.5, 3.9)**	**3.0 (2.2, 4.1)**
	Age 60 and older	**3.4 (2.4, 4.8)**	**1.8 (1.2, 2.6)**	**2.3 (1.7, 3.3)**		**2.4 (1.9, 3.0)**	1.1 (0.8, 1.5)
	Hispanic, Latino, or Spanish^c^	0.6 (0.4, 1.1)	0.7 (0.4, 1.3)	0.6 (0.2, 1.7)	0.6 (0.2, 1.7)	0.7 (0.4, 1.1)	0.7 (0.4, 1.1)
	Non-Hispanic Asian or Pacific Islander	1.5 (0.7, 3.6)	1.6 (0.7, 3.6)	2.1 (0.5, 9.6)	2.3 (0.5, 10.3)	1.7 (0.8, 3.5)	1.8 (0.8, 3.7)
	Non-Hispanic Black	0.6 (0.4, 1.2)	1.0 (0.6, 1.8)	0.6, (0.2, 1.6)	0.7 (0.3, 1.7)	0.7 (0.4, 1.1)	0.9 (0.5, 1.4)
	Other race/ethnicity	0.5 (0.2, 1.3)	0.7 (0.3, 1.8)	0.2 (0.0, 1.0)	0.2 (0.0, 1.4)	0.4 (0.2, 0.9)	0.5 (0.2, 1.2)
Stay home 2019	Program Participant	1.2 (0.9, 1.5)	1.1 (0.8, 1.4)	1.1 (0.8, 1.4)	1.1 (0.8, 1.4)	1.1 (0.9, 1.3)	1.0 (0.8, 1.3)
	Age 60 and older	**1.5 (1.1, 2.1)**	**1.5 (1.0, 2.1)**	1.2 (0.9, 1.5)		**1.2 (1.0, 1.5)**	1.2 (0.9, 1.6)
	Hispanic, Latino, or Spanish	0.7 (0.4, 1.1)	0.7 (0.5, 1.1)	0.5 (0.2, 1.2)	0.5 (0.2, 1.2)	0.7 (0.5, 1.0)	0.7 (0.5, 1.0)
	Non-Hispanic Asian or Pacific Islander	1.4 (0.7, 2.5)	1.4 (0.8, 2.5)	0.4 (0.2, 1.2)	0.4 (0.2, 1.2)	1.0 (0.6, 1.6)	1.0 (0.6, 1.7)
	Non-Hispanic Black	1.0 (0.6, 1.5)	1.1 (0.7, 1.7)	0.5 (0.2, 1.1)	0.5 (0.2, 1.1)	0.8 (0.5, 1.2)	0.9 (0.6, 1.3)
	Other race/ethnicity	0.6 (0.3, 1.2)	0.6 (0.3, 13)	0.3 (0.0, 1.4)	0.3 (0.0, 1.5)	**0.5 (0.2, 0.9)**	0.5 (0.3, 1.0)
Sick at home 2020	Program Participant	**0.3 (0.2, 0.3)**	**0.2 (0.1, 0.3)**	**0.4 (0.3, 0.5)**	**0.3 (0.2, 0.5)**	**0.3 (0.3, 0.4)**	**0.2 (0.2, 0.3)**
	Age 60 and older	**0.6 (0.4, 0.9)**	1.3 (0.8, 2.0)	**0.3 (0.2, 0.4)**		**0.5 (0.4, 0.7)**	1.3 (0.9, 1.8)
	Hispanic, Latino, or Spanish	0.8 (0.5, 1.4)	0.7 (0.4, 1.2)	1.4 (0.6, 3.4)	1.4 (0.6, 3.5)	0.9 (0.6, 1.4)	0.9 (0.5, 1.3)
	Non-Hispanic Asian or Pacific Islander	1.1 (0.6, 2.0)	1.1 (0.6, 2.2)	1.1 (0.4, 3.5)	1.1 (0.3, 3.4)	1.0 (0.6, 1.8)	1.1 (0.6, 1.8)
	Non-Hispanic Black	0.7 (0.4, 1.2)	**0.5 (0.3, 0.9)**	0.9 (0.4, 2.3)	0.8 (0.3, 2.1)	0.7 (0.4, 1.1)	**0.5 (0.4, 0.8)**
	Other race/ethnicity	0.8 (0.3, 2.0)	0.7 (0.3, 1.7)	2.5 (0.4, 15.8)	1.7 (0.3, 10.5)	1.0 (0.4, 2.2)	0.8 (0.4, 1.9)

*OR* odds ratio, *CI* confidence interval; ^a^Adjusted for all other factors listed in table; ^b^Public housing survey had no older adults in the comparison group so age was not included in multivariate model when analyzed separately; ^c^Reference group for race and ethnicity is non-Hispanic White; ^d^ORs with 95% Wald confidence intervals that do not cross 1 are bolded

Both participants and non-participants cited many obstacles to getting an AC in the past. Cost of the unit (62% and 56% respectively), electricity cost (40% and 25% respectively), and cost of proper installation (29% and 24% respectively) were the top barriers (Table 4). “Had air conditioner when I was working. Retired. I had to let it go,” wrote one respondent. Another noted they could “hardly pay my rent.” For public housing residents, the year-round monthly AC surcharge fee was one of the largest barriers to getting an AC in the past. More than one-third (35%, CI: 31, 38) of public housing participants and 41% (CI: 37, 45) of non-participants reported concerns about paying the monthly fee, which ranges from $4 to $10 per AC unit depending on age and income levels and is required by the US Department of Housing and Urban Development (HUD) for resident-owned air conditioners.

**Table 4:** Barriers to getting an air conditioner (AC) in the past among participant and comparison respondents, by housing type, in New York City^a^

	Public housing				Private housing				Pooled			
	Program participants		Comparison group		Program participants		Comparison group		Program participants		Comparison group	
	*n*	% (95% CI)^b^	*n*	% (95% CI)	*n*	% (95% CI)	*n*	% (95% CI)	*n*	% (95% CI)	*n*	% (95% CI)
Pay own electricity at NYCHA	133	22 (19, 25)	29	6 (4, 8)								
Air conditioner (AC) cost	318	53 (49, 57)	230	45 (41, 50)	580	69 (66, 72)	273	69 (65, 74)	898	62 (60, 65)	503	56 (53, 59)
Getting AC properly installed	202	34 (30, 37)	136	27 (23, 31)	224	27 (24, 29)	78	20 (16, 24)	426	29 (27, 32)	214	24 (21, 27)
Increased cost of electricity	130	22 (18, 25)	62	12 (9, 15)	442	52 (49, 56)	164	42 (37, 46)	572	40 (37, 42)	226	25 (22, 28)
Could not go to the store to buy AC	78	13 (10, 16)	36	7 (5, 9)	97	11 (9, 14)	52	13 (10, 17)	175	12 (10, 14)	88	10 (8, 12)
Building installation fees^c^					33	4 (3, 5)	33	8 (6, 11)				
NYCHA or landlord AC surcharge/fee	208	35 (31, 38)	207	41 (37, 45)	26	3 (2, 4)	22	6 (3, 8)	234	16 (14, 18)	229	25 (23, 28)
Cooling assistance application^d^					96	11 (9, 14)	82	21 (17, 25)				
No challenges getting AC the past	95	16 (13, 19)	92	18 (15, 22)	75	9 (7, 11)	23	6 (4, 8)	170	12 (10, 13)	115	13 (11, 15)
Other	33	5 (4, 7)	88	17 (14, 21)	27	3 (2, 4)	34	9 (6, 11)	60	4 (3, 5)	122	14 (11, 16)

^a^Respondents could select more than one answer

^b^Confidence interval

^c^Asked of people not living in public housing only

^d^Difficulty finding or filling out application for cooling assistance. Asked of people not living in public housing only because public housing residents had previously been ineligible for free air conditioners via the Low-Income Home Energy Assistance Program Cooling Assistance Component due to a New York State rule restricting public housing residents’ eligibility. This rule has since been changed

## Discussion

More ACs were installed in both high-heat vulnerability areas and in neighborhoods that were highly impacted by the first wave of COVID-19 in NYC. Survey results indicate that the Get Cool program was successful in helping at-risk individuals stay home safely with AC during a summer of active COVID-19 transmission and extreme heat. Participants were more likely to report staying home in hot weather in 2020 than non-participants, with no difference between groups in 2019 (i.e., pre-intervention). Previous studies have shown that people prefer to be able to stay home during hot weather, but lower-income and non-Latinx Black New Yorkers lack equitable cooling access [9]. Participants in the AC program were also much less likely to report that hot weather made them feel sick in their homes than non-participants, indicating that having the unit helped keep them safe during hot weather. Substantial NYC air quality improvements in spring 2020 related to decreased economic activity may have also contributed to people feeling less sick on hot days [21], but any such impacts would affect the two groups equally so cannot explain lower reports of heat health impacts among participants. The survey also documented the substantial impacts of COVID-19 in the population prioritized by the Get Cool program.

The cost to purchase and run an AC remains a large barrier to equitable access to summer cooling. More than half of participants and non-participants reported that the cost of an AC unit had prevented them from having one in the past. More than 90% of New Yorkers citywide have AC, but there are large inequities by neighborhood, with prevalence ranging from 95% in wealthy neighborhoods to 71% in lower-income areas [22], and lower rates of AC access among Black New Yorkers. In addition, 40% of participants and 25% of non-participants said the additional energy costs were a barrier. This is consistent with previous NYC surveys that also cited increases in electricity bills as a deterrent to AC use [9, 23]. People living in public housing reported that the monthly AC surcharge charged by NYCHA as required by HUD deterred them from getting an AC, even though many residents are highly heat susceptible. Previous studies have shown that inequities in energy security are prevalent, with low-income people and people of color facing a higher energy burden due in part to lack of weatherization, including structural deficiencies and outdated appliances [24, 25].

A relatively small percent of both groups used cooling centers in 2020 and 2019, highlighting the importance of access to home cooling. While the survey did not ask about reasons for cooling center use, some people with AC may go to cooling centers because of concerns about paying the electricity bill [26]. Increasing funding for cooling center programs to allow for more programming to encourage use, extended hours, and advertising in collaboration with community organizations and residents could increase attendance and bolster the utility of these important community resources [27].

While AC helps people to stay home safely in hot weather, it does not replace the need for community supports, including for mental health and other social services. Social isolation during social distancing and other stressors from the pandemic, such as loss of a job, illness, or loss of friends or loved ones, may have exacerbated or increased the existing burden of mental health problems. In addition to the prevalence of chronic conditions, there were high levels of anxiety, depression, and mental health conditions reported among all households surveyed. In comparison, the NYC Community Health Survey, a representative telephone survey of NYC adults, measured current prevalence of depression at 9.3% (8.1, 9.8) citywide in 2017 [28]. While this was an individual measure and we asked about household prevalence of mental health conditions, the average of 26% of households across both groups reporting a mental health condition paints a picture of the isolation and mental stress that respondents experienced during and following the first COVID-19 wave in NYC. This underscores the importance of coupling interventions to increase home safety and comfort with social connectedness programs.

Our analysis had several limitations. For the map of installations, denominator data that matched eligibility criteria exactly was not available. However, mapping installations against the number of older adults living below the federal poverty line, although a lower income threshold than required by the Get Cool program, provides a visualization of how many ACs were delivered in areas with larger numbers of resource-limited older adults. In addition, the heat vulnerability index and TRIE area designations include measures of limited financial resources. Because people took the survey in late 2020 and early 2021, there may have been recall bias and confusion about questions that asked about “this summer (2020)” versus “last summer (2019),” despite inclusion of the year in all questions. In addition, more than a third of program participants reported having an AC in 2019. These respondents may have been confused by the question timeframe and wording, which asked about which rooms in the home had AC “last summer (2019),” since the survey was mailed in fall 2020 and responses collected into early 2021, the next calendar year. In addition, AC units may have broken, or survey respondents may have moved to housing without AC prior to 2020.

Participants who were aware that the survey was asking about their new AC may have been more likely to report using it, potentially leading to an over-estimation of benefits. However, the magnitude of difference between the participant and comparison group in staying home and not feeling ill is unlikely to be solely explained by this factor. Differences between our participant and non-participant groups may also affect comparability. There may have been confounding by age because by necessity our comparison group skewed younger and reported a higher, though still minimal, prevalence of going to a workplace. Older adults may have been more likely to follow social distancing recommendations out of fear of contracting COVID-19 than younger adults, also affecting comparability of the groups. About 25% of our comparison group, however, was aged 60 or older, and younger and older non-participants had similar rates of staying home in 2019 and 2020. The comparison group also differed from the participant group by race and ethnicity, with fewer Latinx respondents and more Black respondents.

Because we did not aim to make the survey representative of any group beyond Get Cool participants, we did not weight survey responses to be representative of a larger population. Participants of the Get Cool program itself were largely enrolled based on participation in other safety-net programs, with the exception of the DOHMH community- and faith-based organization partner enrollment. Previous research has shown that people linked to services may be more likely to receive other types of services [29]. The Get Cool program and this evaluation both suffered from a general limitation of government programs that recruit from people connected to existing programs—they likely missed New Yorkers who would have benefitted from the service and cannot estimate outcomes for that population.

## Conclusions

The emergency distribution and installation of free air conditioners helped participants stay home safely in summer 2020. While Get Cool was launched as a joint COVID-19 and heat emergency response measure, program outcomes are relevant for heat adaptation with or without active COVID-19 transmission. AC access is critical to achieving climate justice as cities continue to warm, and public health crises persistently impact the same heat-vulnerable communities that have endured decades of systemic racism and disinvestment.

AC access could be improved by substantially improving the reach, decreasing the application burden, and increasing funding levels of existing cooling assistance programs, such as the Home Energy Assistance Program (HEAP). Currently, HEAP in New York provides a small amount of cooling assistance each year by funding the purchase of an AC but does not provide summer utility assistance. Given that NYC has one of the largest energy cost burdens in the country for low-income residents [30], HEAP and other energy assistance programs also should provide utility subsidies so people can afford to run ACs in summer, in line with the life-saving supports instituted in the past to ensure winter heat. In addition, the City should explore regulations to require landlords to provide access to working AC and prohibit additional surcharges for use.

Given the huge public health challenges posed by climate change in cities like NYC, increased AC access needs to go hand-in-hand with other interventions. Public AC access programs should, at minimum, prioritize providing efficient models and properly air-sealing units when installed. Additional interventions should include initiatives like increasing funding for energy efficiency and weatherization programs, ramping up community solar programs to help ease energy costs, increasing funding for cooling centers and greening in high heat-risk areas, and funding community-led resilience programs. Heat waves and other climate change hazards exacerbate existing inequities, and interact with other public health emergencies, as demonstrated by the COVID-19 pandemic, requiring not only emergency but also structural and sustainable solutions.

## Supplementary Information

Below is the link to the electronic supplementary material.

Supplementary file1 (DOCX 17.2 KB)

Supplementary file2 (PDF 178 KB)

Supplementary file3 (PDF 180 KB)
