# Supplementary material for: Extreme Heat and COVID-19 in New York City: An Evaluation of a Large Air Conditioner Distribution Program to Address Compounded Public Health Risks in Summer 2020
Source: J Urban Health. 2023 Feb 9;100(2):290–302. doi: 10.1007/s11524-022-00704-9 (PMC9910776; doi:10.1007/s11524-022-00704-9)
Supplement: Supplementary file 1 — Supplementary file1 (DOCX 17.2 KB) [file 11524_2022_704_MOESM1_ESM.docx]

| Supplemental Table 1. Get Cool Air Conditioner (AC) Installation Rates by Heat Vulnerability Index (HVI), Task Force for Racial Inclusion and Equity (TRIE) area, and older adult neighborhood poverty level. | | | | | |
| --- | --- | --- | --- | --- | --- |
|  | **2020 AC installations** | **Rate per 1,000 adults 60 and older** | **Rate ratio** |  |  |
| **Heat Vulnerability Index level** |  |  |  |  |  |
| 1-3 (lower risk) | 20,478 | 20.4 | reference |  |  |
| 4 (higher risk) | 19,722 | 47.7 | 2.3 |  |  |
| 5 (highest risk) | 32,409 | 94.1 | 4.6 |  |  |
|  |  |  |  |  |  |
| **Task Force for Racial Inclusion and Equity area** |  |  |  |  |  |
| No | 17,124 | 18.1 | reference |  |  |
| Yes | 55,485 | 68.1 | 3.8 |  |  |
|  |  |  |  |  |  |
| **Percent of older adults (60+) living below the federal poverty line** | | |  |  |  |
| <10% | 4,242 | 10.5 | reference |  |  |
| 10 to <20% | 18,895 | 28.6 | 2.7 |  |  |
| 20 to <30% | 27,240 | 63.1 | 6.0 |  |  |
| 30 to 100% | 22,232 | 119.6 | 11.4 |  |  |
| Note: Installation numbers and rate numerators based on NYC Emergency Management Get Cool program AC installation data. HVI and TRIE rate denominators based on NYC DOHMH population estimates for modified zip code tabulation areas, modified from US Census Bureau intercensal population estimates, for all adults aged 60 years and older in 2019. TRIE neighborhoods were designated by NYC in 2020 as those most impacted by COVID-19 with a high percentage of other health and socioeconomic inequities. The HVI is based on a statistical model that uses social and environmental factors to estimate heat-related mortality risk across NYC neighborhoods. Older adults in poverty rate denominators based on U.S. Census Bureau. 2015-2019 American Community Survey 5-year Public Use Microdata Samples for adults 60 and older for whom poverty status could be determined. | | | |  |  |
|  |  |  |  |  |  |
|  |  |  |  |  |  |
|  |  |  |  |  |  |
|  |  |  |  |  |  |
