# Supplementary material for: Extreme Heat and COVID-19 in New York City: An Evaluation of a Large Air Conditioner Distribution Program to Address Compounded Public Health Risks in Summer 2020
Source: J Urban Health. 2023 Feb 9;100(2):290–302. doi: 10.1007/s11524-022-00704-9 (PMC9910776; doi:10.1007/s11524-022-00704-9)
Supplement: Supplementary file 2 — Supplementary file2 (PDF 178 KB) [file 11524_2022_704_MOESM2_ESM.pdf]

Supplemental material: Survey questions (unformatted) for private housing residents, non-participants

1. If you received or bought a working air conditioner this summer (2020), what month did you have it installed?
   - Before June
   - June
   - July
   - August
   - September
   - I do not remember
   - I have a working air conditioner but did not receive or buy it this summer
   - I do not have an air conditioner

2. In which room(s) do you currently have a working air conditioner?
   Choose all that apply
   - Living room or main living area
   - Bedroom
   - Other room
   - I do not have a working air conditioner

3. In which room(s) did you have a working air conditioner in your home last summer (2019)?
   Choose all that apply
   - Living room
   - Bedroom
   - Other room
   - I did not have a working air conditioner

4. This summer (2020), how often did you use your air conditioner when you were at home during very hot weather?
   - Always
   - Most of the time
   - About half the time
   - Less than half the time
   - Never
   - I do not have an air conditioner

5. Last summer (2019), how often did you use your air conditioner when you were at home during very hot weather?
   - Always
   - Most of the time
   - About half the time
   - Less than half the time
   - Never

- I did not have an air conditioner

6. This summer (2020), if your household used an air conditioner, at what time of day did you use your air conditioner most?

   - Morning
   - Afternoon
   - Evening
   - Overnight
   - All day and all night
   - I did not use an air conditioner

7. If you have an air conditioner but didn't use it much, what prevented you from using it? *Choose all that apply*

   - Cost of the electric bill
   - Fees my building charges for air conditioner use
   - Cost of the New York City Housing Authority (NYCHA) air conditioner fee (for NYCHA residents only)
   - Did not work
   - Cost of repairs
   - Noise
   - Medical reasons
   - Worried it will be too cold or uncomfortable
   - Preferred other methods for keeping my home cool
   - Nothing prevented me from using my air conditioner
   - I did not have an air conditioner
   - Other, please explain:

8. This summer (2020), which of the following methods did you use to cool your home?
   Choose all that apply
   - Fan(s)
   - Open windows
   - Air conditioner(s)
   - Nothing
   - Other, please explain _______________

9. Last summer (2019), what did your household do to cool your home?
   Choose all that apply
   - Fan(s)
   - Open windows
   - Air conditioner(s)
   - Other, please explain _______________

10. This summer (2020), where did you go most often to cool off in very hot weather?

Choose up to 3 answers

- Stayed at home
- Visited a neighbor/friend/family member's house
- Went to a work location
- Went to a business (pharmacy, grocery store, barber shop, etc.)
- NYC "Cool Street" program (street closed to cars)
- Went somewhere else outside (beach, park, stoop)
- Went to a City-designated cooling center (such as a senior center, community center or public library)
- Went to a church, temple, mosque or other place of worship
- Other, please explain __________

11. Last summer (2019), where did you go most often to cool off in very hot weather?

Choose up to 3 answers

- Stayed at home
- Visited a neighbor/friend/family member's house
- Went to a work location
- Went to a business (pharmacy, grocery store, barber shop etc.)
- Went somewhere else outside (beach, park, stoop)
- Went to a City-designated cooling center (senior center, community center or public library)
- Went to a church, temple, mosque or other place of worship
- Other, please explain __________

12. This summer (2020), if you stayed home during very hot weather, what were the main reasons?

Choose up to top 3 answers

- Home was comfortable
- Concerned about COVID/social distancing
- Concerned about safety in the community
- Childcare responsibilities
- Working from home
- Nowhere else to go
- Limited transportation
- Had difficulty moving or walking
- Not applicable/I did not stay home
- Other, please explain _________

13. Last summer (2019), if you stayed home during very hot weather, what were the main reasons?

Choose up to 3 answers

- Home was comfortable
- Concerned about safety in the community
- Childcare responsibilities
- Working from home
- Nowhere else to go
- Limited transportation
- Had difficulty moving or walking

- Not applicable/I did not stay home
- Other, please explain ____________

14. How likely would you be to go to a cooling center (such as a public library, senior center or community center)?
    - Extremely likely
    - Very likely
    - Somewhat likely
    - Not very likely
    - Not likely at all

15. What was your primary source of warnings about extreme heat this summer (2020)?
    - NYC government announcement (for example, Notify NYC text messages or emails)
    - Church, mosque, synagogue or another religious site
    - TV (news or weather report)
    - Radio
    - Internet/phone app/social media
    - Newspaper
    - Word of mouth/ friend/family
    - Community group
    - Other
    - Did not see or hear any extreme heat warnings

16. What have been your biggest challenges in getting an air conditioner in the past?
    Choose all that apply
    - The AC unit was too expensive/I couldn't afford it
    - Getting the air conditioner unit properly installed
    - Concerns about the increased cost of electricity
    - Could not go to the store to buy the air conditioner
    - Fees my building charges for installation
    - NYCHA or landlord air conditioner surcharge/fee
    - Difficulty finding or filling out the application for cooling assistance
    - No challenges getting an air conditioner unit in the past
    - Other, please explain ________________________

17. This summer (2020), did holding back on electricity use (due to concerns about the bill) affect any of the following? *Choose all that apply.*
    - Your sleep quality
    - Your stress levels or mood
    - Your health conditions (for example asthma or hypertension)
    - Your willingness to invite friends or family over to your house
    - Your decisions about food purchases (for example, I used less electricity so I could buy food)
    - Your decisions about medicine purchases (for example, I used less electricity so I could buy medicine)
    - This did not affect me

18. How has COVID-19 affected your household? *Choose all that apply.*
   - o A household member lost their job
   - o A household member was diagnosed with COVID-19
   - o A household member was hospitalized due to COVID-19
   - o A household member passed away from COVID-19
   - o I have had trouble with providing food for family
   - o I have had trouble with paying my rent or mortgage
   - o I have had trouble with providing/affording childcare
   - o I have had trouble with helping my children with their schooling
   - o It did not affect my household
   - o Other, please explain _________________

19. In the past 12 months (Summer 2019- Summer 2020), have you ever had trouble paying your electric and gas bills in full and on time?
   - o Yes
   - o No
   - o Not applicable/I do not have an electric/gas bill

20. In the past 12 months (Summer 2019- Summer 2020), have you received a disconnection notice for your electricity or gas services?
   - o Yes
   - o No
   - o Not applicable/I do not have an electric/gas bill

21. In the past 12 months (Summer 2019- Summer 2020), have your electricity or gas services been shut off due to non-payment?
   - o Yes
   - o No
   - o Not applicable/I do not have an electric/gas bill

22. Has your current household ever applied for energy assistance services to help pay for your utility bills?

   Choose all that apply
   - o Yes, I applied to the Home Energy Assistance Program (HEAP) for help with purchasing an air conditioner
   - o Yes, I applied to HEAP for help with my winter heating costs
   - o Yes, I applied to another program to help with electric/gas bills
   - o No
   - o Not sure

23. THIS summer (2020), do you think the hot weather has made you or members of your household feel sick or worsened existing health conditions while at home?

- Yes
- No
- Not sure

Heat related illness can include nausea, cramps, dry mouth, dizziness, fainting, fatigue, rapid heartbeat or hallucinations.

24. Do you or anyone in your household have any of the following health conditions?

Choose all that apply

- Diabetes
- Hypertension (high blood pressure)
- Cardiovascular conditions (heart conditions)
- Overweight or obese
- Asthma or chronic obstructive pulmonary disease (COPD)
- Physical mobility hardships
- Chronic kidney disease
- Need to use electric medical equipment
- Cognitive impairments
- Anxiety, depression or other mental health conditions
- None
- Prefer not to say

25. Would you say that in general your health is excellent, very good, good, fair or poor?

- Excellent
- Very good
- Good
- Fair
- Poor
- Do not know

26. How old are you?

- 18 – 29
- 30 – 39
- 40 – 49
- 50 – 59
- 60+

27. How many people, including yourself, live in your household? Please list how many of your household members fall in each age range listed below.

Please include yourself and all household members in this section

- Total number of household members, including you. __________
- Number of children 5 years old and under. __________
- Number of children from 6 to 18 years old. __________

- Number of adults from 19 to 59 years old. __________
- Number of adults from 60 to 69 years old. __________
- Number of adults from 70 years and above. __________

28. Which of the following best represents your race and/or ethnicity.

Choose all that apply

- Black or African American
- Hispanic, Latino or Spanish
- Middle Eastern or North African
- White
- Asian or Pacific Islander
- American Indian, Native, First Nations, Indigenous Peoples of the Americas or Alaska Native
- Other, please specify:
- Prefer not to say

29. If you added together the yearly income of all the members of your family living at home last year, would the total be…

Select one

- Less than $20,000
- $20,000 to less than $40,000
- $40,000 to less than $60,000
- $60,00 to less than $80,000
- $80,000 or more
- Do not know
